# Genome Sequence of a Podovirus (AAPEc6) Isolated from Wastewater in New Zealand That Infects *Escherichia coli* O45:H10

**DOI:** 10.1128/genomeA.00462-17

**Published:** 2017-08-03

**Authors:** Judith Nonis, Aruni Premaratne, Craig Billington, Arvind Varsani

**Affiliations:** a School of the Biological Sciences, University of Canterbury, Christchurch, New Zealand; b Institute of Environmental Science & Research Ltd., Christchurch, New Zealand; c Structural Biology Research Unit, Department of Clinical Laboratory Sciences, University of Cape Town, Cape Town, South Africa; d The Biodesign Center for Fundamental and Applied Microbiomics, Center for Evolution and Medicine, School of Life Sciences, Arizona State University, Tempe, Arizona, USA

## Abstract

Bacteriophages are ideal candidates for pathogen biocontrol to mitigate outbreaks of prevalent foodborne pathogens, such as *Escherichia coli*. We identified a bacteriophage (AAPEc6) from wastewater that infects *E. coli* O45:H10. The AAPEc6 genome sequence shares 93% identity (with 92% coverage) to enterobacterial phage K1E (*Sp6likevirus*) in the *Autographivirinae* subfamily (*Podoviridae*).

## GENOME ANNOUNCEMENT

Bacteriophages are being used in the food industry to prevent bacterial contamination (1). As part of an ongoing effort to identify phages for potential use for biocontrol of pathogenic bacteria, especially in the food industry, wastewater samples were sourced from a local dairy company. The samples were centrifuged at 3,000 × *g*, the supernatant was filtered through a 0.22-µm filter, and 0.1 ml of filtrate was added to soft overlay nutrient agar plates containing log-phase *Escherichia coli*. Plaque formation was checked following incubation at 37°C for 18 to 24 h. We screened a panel of 20 strains of *E. coli*, which contain representatives of all 7 serotypes declared as adulterants on meat by the U.S. Food Safety and Inspection Service (FSIS), and found clear plaques on lawns of *E. coli* strain NZRM 1345 (also known as NCTC9045). The *E. coli* serotype is O45:H10 and was originally isolated from Copenhagen, Denmark, in 1952. Phage AAPEc6 was isolated from this host following three rounds of purification.

A high-titer stock of AAPEc6 *E. coli* phage was prepared by the soft agar overlay method, and 0.2 ml of the ~10^11^ PFU/ml titer stock was used to extract viral DNA using the High Pure viral nucleic acid kit (Roche, USA). The purified DNA was sequenced on an Illumina HiSeq 2500 (Illumina, USA) platform at Macrogen, Inc. (Hong Kong). The paired-end reads were *de novo* assembled using ABySS version 1.9 (2). In the largest contig, 45,086 bp, a terminal repeat region of 261 bp was identified, and a BLASTn analysis of the linear contig without the terminal repeat (44,825 bp) revealed that the *de novo*-assembled genome of AAPEc6 *E. coli* phage is most closely related to genomes of phages in the *Autographivirinae* subfamily (family *Podoviridae*) with four genera (*Kp34virus*, *Phikmvvirus*, *Sp6virus*, and *T7virus*) in the order *Caudovirales*. BLASTn analysis of AAPEc6 *E. coli* phage shows that it is most closely related to the enterobacterial phage K1E (accession no. AM084415; *Sp6likevirus*) (3), with 93% pairwise nucleotide identity with 92% coverage; therefore, it is a member of the *Sp6likevirus* genus. Posterior mapping of the paired-end reads using Bowtie (4) indicated that 26,010,817 reads mapped to the genome, with a minimum depth of coverage of 70,000×. Fifty-two open reading frames were identified in the genome of AAPEc6 *E. coli* phage that were organized similarly to those in enterobacterial phage K1E. Of these, 22 encode proteins of known function (ranging in size from 64 to 1,102 residues) which are homologues to those encoded by most other members of the *Autographivirinae* subfamily. Of all the viruses in the *Autographivirinae* subfamily, a lyase protein is only encoded by AAPEc6 *E. coli* phage, *Escherichia* virus K1-5 (accession no. AY370674), and a partial genome sequence *Escherichia* virus K5 (Y10025) sharing >98% amino acid identity.

In summary, here we report the genome sequence of a bacteriophage, AAPEc6, isolated from wastewater samples in New Zealand, which is a *Sp6likevirus* in the *Autographivirinae* subfamily (family *Podoviridae*) that infects *E. coli* O45:H10. AAPEc6 may have potential for biocontrol of *E. coli* in combination with other phages.

### Accession number(s).

The complete genome sequence of the AAPEc6 *E. coli* phage has been deposited at GenBank under the accession no. KX279892.
